# Constructal Optimizations for Heat and Mass Transfers Based on the Entransy Dissipation Extremum Principle, Performed at the Naval University of Engineering: A Review

**DOI:** 10.3390/e20010074

**Published:** 2018-01-19

**Authors:** Lingen Chen, Qinghua Xiao, Huijun Feng

**Affiliations:** 1 Institute of Thermal Science and Power Engineering, Naval University of Engineering, Wuhan 430033, China; 2 Military Key Laboratory for Naval Ship Power Engineering, Naval University of Engineering, Wuhan 430033, China; 3 College of Power Engineering, Naval University of Engineering, Wuhan 430033, China

**Keywords:** constructal theory, entransy dissipation extremum principle, heat conduction, convective heat transfer, mass transfer, generalized thermodynamic optimization

## Abstract

Combining entransy theory with constructal theory, this mini-review paper summarizes the constructal optimization work of heat conduction, convective heat transfer, and mass transfer problems during the authors’ working time in the Naval University of Engineering. The entransy dissipation extremum principle (EDEP) is applied in constructal optimizations, and this paper is divided into three parts. The first part is constructal entransy dissipation rate minimizations of heat conduction and finned cooling problems. It includes constructal optimization for a “volume-to-point” heat-conduction assembly with a tapered element, constructal optimizations for “disc-to-point” heat-conduction assemblies with the premise of an optimized last-order construct and without this premise, and constructal optimizations for four kinds of fin assemblies: T-, Y-, umbrella-, and tree-shaped fins. The second part is constructal entransy dissipation rate minimizations of cooling channel and steam generator problems. It includes constructal optimizations for heat generating volumes with tree-shaped and parallel channels, constructal optimization for heat generating volume cooled by forced convection, and constructal optimization for a steam generator. The third part is constructal entransy dissipation rate minimizations of mass transfer problems. It includes constructal optimizations for “volume-to-point” rectangular assemblies with constant and tapered channels, and constructal optimizations for “disc-to-point” assemblies with the premise of an optimized last-order construct and without this premise. The results of the three parts show that the mean heat transfer temperature differences of the heat conduction assemblies are not always decreased when their internal complexity increases. The average heat transfer rate of the steam generator obtained by entransy dissipation rate maximization is increased by 58.7% compared with that obtained by heat transfer rate maximization. Compared with the rectangular mass transfer assembly with a constant high permeability pathway (HPP), the maximum pressure drops of the element and first-order assembly with tapered HPPs are decreased by 6% and 11%, respectively. The global transfer performances of the transfer bodies are improved after optimizations, and new design guidelines derived by EDEP, which are different from the conventional optimization objectives, are provided.

## 1. Introduction

Heat and mass transfers exist widely in nature and engineering. Some heat and mass transfer enhancement problems exist, such as how to effectively dissipate heat flow that is generated in an electronic device, strengthen convective heat transfer in a confined space [1,2,3,4,5,6], and scatter mass flow generated in a mass source [7,8], etc. However, a uniform theory to guide heat and mass transfer enhancements is lacking.

Bejan [9] found the constructal law and put forward the constructal theory [10,11,12,13,14,15] in 1996. The first application of this law was the heat conduction optimization of an electronic device [9]. The constructal law is a law of physics, which is far more general than “minimum entropy generation”. It can be described as follows [9]: “For a finite-size flow system to persist in time (to live), it must evolve such that it provides greater and greater access to the currents that flow through it.” In recent years, various flow systems have been studied and optimized based on constructal theory. Gradually, constructal theory has become a useful tool to solve the optimization problems in various fields.

Various performance indexes have been taken as optimization objectives in heat and mass transfer optimizations. To reflect the essence of the heat transfer process, based on the analogy between heat conduction and electrical conduction processes, Guo et al. [16,17] proposed a new physical quantity called “entransy” and the entransy dissipation extremum principle (EDEP). Entransy is a physical quantity to describe the total heat transfer ability of an object and can be expressed as $E_{Vh}$:

(1) $$E_{Vh}=Q_{Vh}T/2$$

where $Q_{Vh}$ is the constant volume of heat and *T* is the temperature of the object. It was called the heat transport potential capacity and phenomenologically corresponded to energy in the electrical capacitor.

Heat quantity as a kind of energy is conserved in the heat transfer process but the heat transfer ability of the object is dissipated. That is, the entransy or heat transport potential capacity should be dissipated. In the heat transfer process, the dissipation rate of entransy per unit time and volume is named as the entransy dissipation function $\phi_{h}$, which can be expressed as

(2) $$\phi_{h}=-\dot{q}\cdot \nabla T=k{\left(\nabla T\right)}^{2}$$

where $\dot{q}$ is the heat flux vector, k is thermal conductivity, and ∇T is the temperature gradient. From Equation (2), the entransy dissipation rate ${\dot{E}}_{Vh\phi }$ in the whole volume of an object is

(3) $${\dot{E}}_{Vh\phi }=\int_{V}\phi_{h}dV$$

where V is the volume of the object.

Using the concept of entransy, EDEP, which can guide heat transfer optimization, is deduced as follows [16,17]: “For a fixed boundary heat flux, the heat conduction process is optimized when the entransy dissipation is minimized (minimum temperature difference); while for a fixed boundary temperature, the heat conduction process is optimized when the entransy dissipation is maximized (maximum heat flux).”

The optimizations based on the maximum potential difference minimization can only reduce the maximum potential of a body and reflect its local transfer performance. To improve the global transfer performance of transfer bodies, the entransy theory [18,19,20,21] has been combined with the constructal theory and a series of constructal optimizations have been conducted. This paper will summarize the work on heat conduction and finned cooling problems, cooling channel and steam generator problems, and porous medium mass transfer problems as performed at the Naval University of Engineering. Comparisons of the optimal results obtained by different optimization objectives and assembling methods have been conducted. The contribution of this review is a summary of the benefits and findings of constructal optimization works for heat and mass transfers based on the entransy theory. The global transfer performances of the transfer bodies are improved after optimizations. The new design guidelines derived by EDEP, which are different from the conventional optimization objectives, are provided.

## 2. Constructal Optimizations Based on the Entransy Dissipation Extremum Principle

Based on the constructal theory and entransy theory, heat conduction and finned cooling problems, cooling channel and steam generator problems, and porous medium mass transfer problems have been investigated. Comparisons of the optimal results obtained by the extremes of entransy dissipation and other optimization objectives have been conducted.

### 2.1. Heat Conduction and Finned Cooling Problems

Bejan [9] introduced the constructal theory into structure optimization of a rectangular electronic device and reduced its peak temperature with the assumption of linear heat current distribution. Ghodoossi and Egrican [22] found that the optimization result was not the same as that derived in Reference [9] when an exact solution was adopted in the constructal optimization, and Wu et al. [23] gave further reason for the result difference by using new equivalent thermal conductivity. The heat conduction problems were further extended to rectangular bodies with discrete variable cross-sections [24], X- [25], V- [26], “+” [27], I- [28], and T-shaped [29] high conductivity channels (HCCs), non-uniform heat generation [30] and triangular [31,32] and disc-shaped [33,34] bodies of different scales. Neagu and Bejan [35] and Wu et al. [36] tried to improve the heat conduction performances (HCPs) of rectangular bodies by using a tapered structure and a global constructal optimization method, and reduced the maximum thermal resistances (MTRs) of the bodies by 33% and 45%, respectively. Chen et al. [37] further re-optimized the rectangular body built in Reference [9] by taking the minimum entransy dissipation rate (EDR) as the optimization objective and found that the optimal constructs of rectangular bodies derived by different optimization objectives were different when the heat current distribution along the HCC was not linear. Henceforth, heat conduction constructal optimizations based on different optimization objectives [38,39,40,41,42,43] have become a hot topic for constructal investigators.

Fins with convective heat transfer area useful way to dissipate heat from a heat generating body [44]. Bejan and Almogbel [45] and Almogbel [46] built the T-, Y-, umbrella-, and tree-shaped fin models, and minimized the MTRs of these fins by optimizing their external shapes based on the constructal theory. Xie et al. [47] and Lorenzini and Rocha [48] further investigated the T- and Y-shaped fins based on the finite element method; they also built T-, Y- [49], and twice level Y-shaped [50] fins to improve heat transfer performances (HTPs). The analytical solutions of the fins were verified by the numerical solutions and the result showed that the MTR reduction of Y-shaped fin reached 36.37%. Moreover, the cylindrical pin-fin [51,52] and helm-shaped fin [53,54] were also optimized based on different optimization objectives and different guidelines for the fin designs were provided.

**Figure 1:**
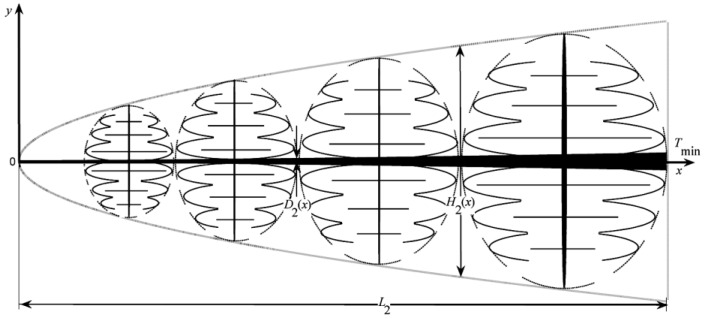
Tapered second-order assembly [55].

**Figure 2:**
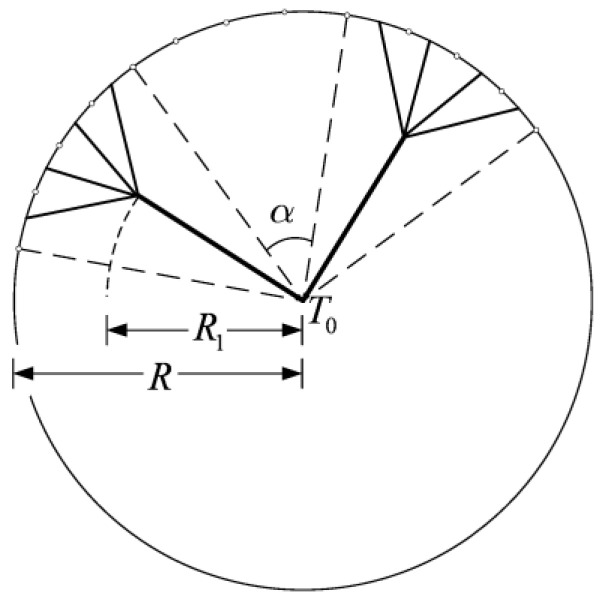
First-order assembly of a disc [56].

**Figure 3:**
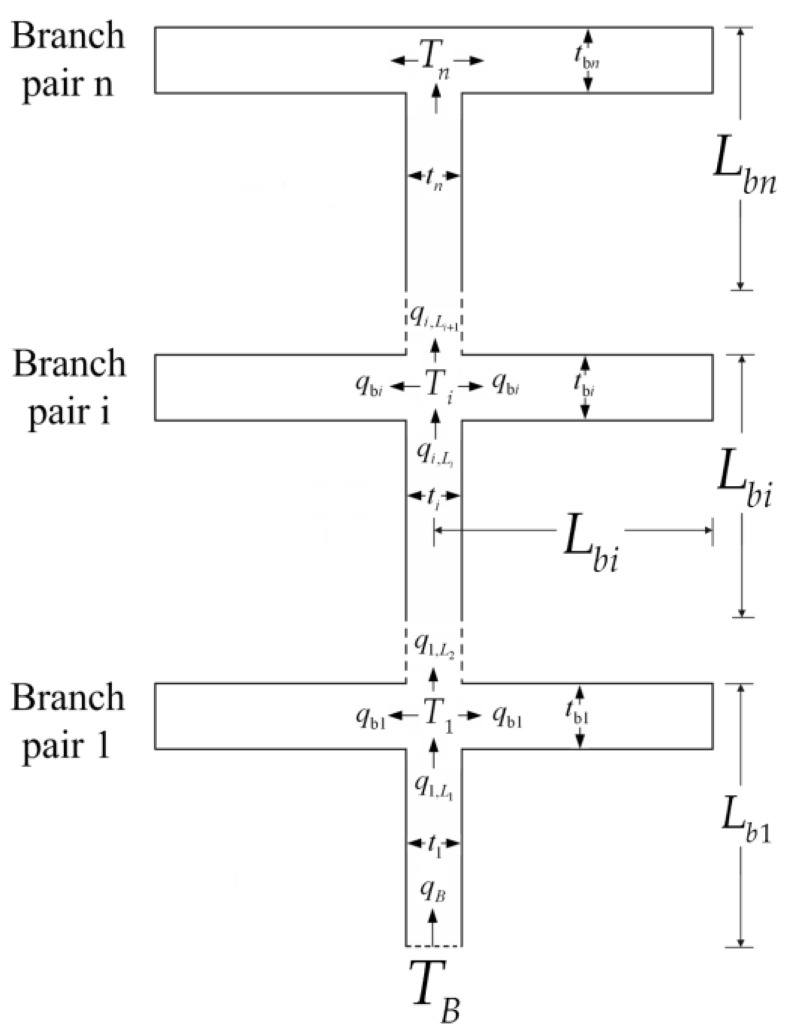
Tree-shaped assembly of fins [61].

A tapered assembly with tree-shaped HCCs is shown in Figure 1 [55] where $D_{2}$, $H_{2}$, and $L_{2}$ are the width of a second-order HCC, and height and length of a second-order assembly, respectively. The heat is uniformly generated in the low conductivity material and the volumes of the HCC and tapered assembly are fixed. The minimum EDR was taken as the optimization objective. Constructal optimization of the tapered assembly was implemented and the external and internal structures of the tapered assembly were optimized. The results obtained were compared with those obtained based on the maximum temperature difference (MTD) minimization, and different optimal constructs based on different optimization objectives were obtained. Similar to the work of a tapered assembly, the “disc-to-point” heat-conduction problems (Figure 2) with the premise of an optimized last-order construct [56] and without this premise [57] were also investigated. In Figure 2, $R_{1}$, R, α, and $T_{0}$ are the radii of the central disc and first-order disc, the angle of the first-order sector and the outlet temperature, respectively. Moreover, constructal optimizations for four kinds of fins (i.e., T-shaped [58], Y-shaped [59], umbrella-shaped [60] and tree-shaped (Figure 3) [61] fins) were also implemented based on EDR minimization. In Figure 3, $t_{bi}$, $t_{i}$, $L_{bi}$, $q_{bi}$, and $q_{L,Li}$ are the branch thickness, main channel width, element length, and heat flow rates in the branch and main channel, respectively.

**Figure 4:**
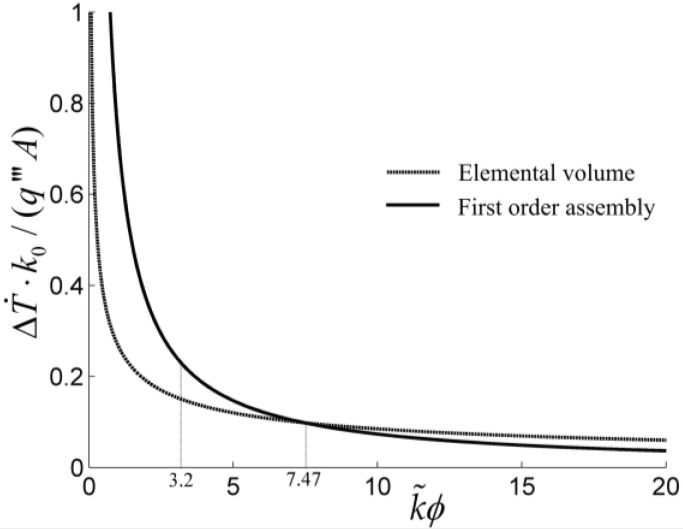
Mean temperature differences for the element and first-order assembly [55].

The results showed that the optimal constructs of the tapered assembly, disc-shaped assembly, and the four kinds of fins discussed, based on the minimizations of EDR and MTD, are different. The optimal constructs based on EDR minimization decrease the mean heat transfer temperature differences (MHTTDs) and improve their HCPs. For the tapered assembly, the MHTTD is not always decreased when the internal complexity of the assembly is increased (Figure 4), but an optimal order of the assembly exists that leads to a minimum MHTTD. In Figure 4, ΔT, $k_{0}$, q‴, A, $\tilde{k}$, and ϕ are the temperature difference of the assembly, thermal conductivity of the heat generation area, volumetric heat generation rate, area of the assembly, thermal conductivity ratio, and volume fraction of HCC, respectively.

**Figure 5:**
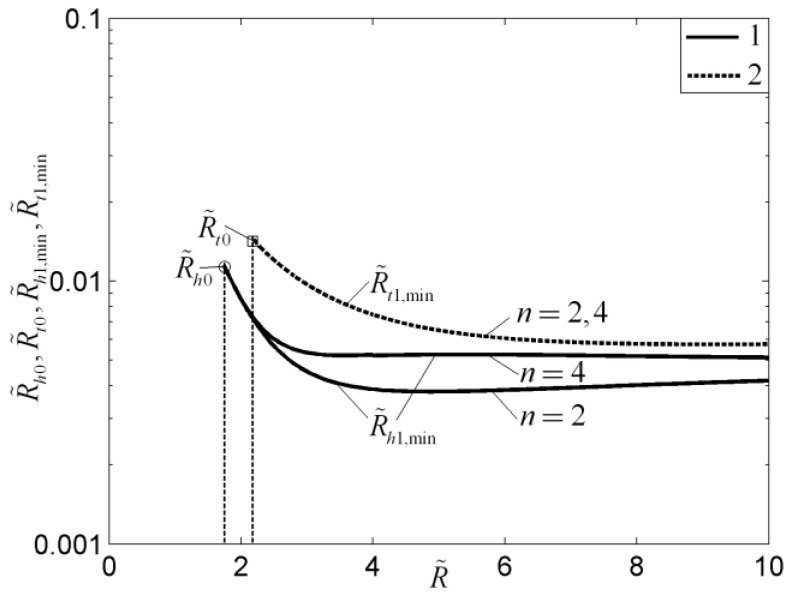
Optimal results of a disc-shaped assembly based on different optimization objectives [56].

**Figure 6:**
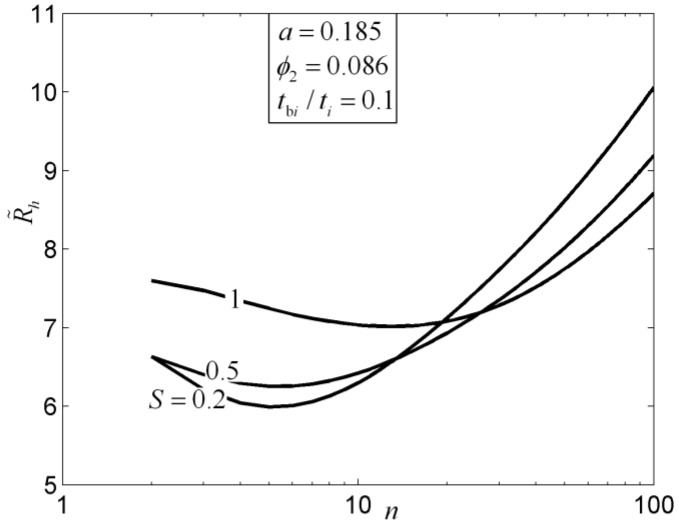
Characteristic of equivalent thermal resistance versus branch number [61].

For the disc-shaped assembly, the critical radii based on the minimizations of EDR and MTD, which determine whether the radial-patterned disc or tree-shaped disc is adopted, are different (Figure 5). In Figure 5, $\tilde{R}$, ${\tilde{R}}_{t0}$, ${\tilde{R}}_{h0}$, ${\tilde{R}}_{t1}$, ${\tilde{R}}_{h1}$, and n are the dimensionless radius of the disc, dimensionless MTR of the elemental disc, dimensionless equivalent thermal resistance (ETR) of the elemental disc, dimensionless MTR of the first-order disc, dimensionless ETR of the first-order disc, and branch number, respectively. Moreover, the subscript “min” represents the minimum value of the dimensionless thermal resistance. The HCP of the disc-shaped assembly could be further improved by releasing the premise of an optimized last-order construct. For the fins, the global HTPs of Y-shaped and tree-shaped fins are better than that of the T-shaped fin. The global HTP of the tree-shaped fin is not always better when the internal structure becomes more complex (Figure 6). In Figure 6, a, $\phi_{2}$, n, and ${\tilde{R}}_{h}$ are the convective heat transfer parameter, fin volume fraction, branch number, and dimensionless ETR, respectively.

### 2.2. Cooling Channel and Steam Generator Problems

Another effective heat dissipation method is the convective heat transfer in the cooling channel. Bejan and Errera [62] inserted a cooling channel into a rectangular heat generating body and minimized the MTR and overall flow resistance (OFR) by optimizing the internal and external shapes of the body, respectively. Ordonez [63] minimized the MTD of a cuboid heat generating body by using the asymptote intersection principle and obtained the optimal space between the adjacent cooling channels. Feng et al. [64] conducted a constructal design of a disc-shaped heat generating body with specified pumping power and obtained the minimum MTR and critical radius of a first-order disc-shaped body. Moreover, Xie et al. [65,66], Song et al. [67], and Adewumi et al. [68,69] further built heat generating models with multistage bifurcation [65], Y- [66], wavy-shaped [67], pin-fin inserted [68], and temperature-dependent fluid property [69] cooling channels, and obtained better and more actual HTPs of the heat generating bodies.

Opposite to the heat dissipation processes with HCCs or cooling channels, the heat should be absorbed by the steam generator as much as possible. Kim et al. [70,71] maximized the heat transfer rate (HTR) of a steam generator with fixed volumes of a steam generator and heat transfer tubes and obtained the optimal adjacent space and numbers of the riser and downcomer tubes. Norouzi et al. [72] conducted constructal design of a heat recovery steam generator and minimized the entropy generation number of a steam generator by optimizing the tube radii of the economizer, evaporator, and superheater, respectively. Mehrgoo and Amidpour [73] further optimized entropy generation performance of a dual pressure heat recovery steam generator and found that the total volume of the steam generator had its optimum value and the heat conductance of the evaporator was big.

**Figure 7:**
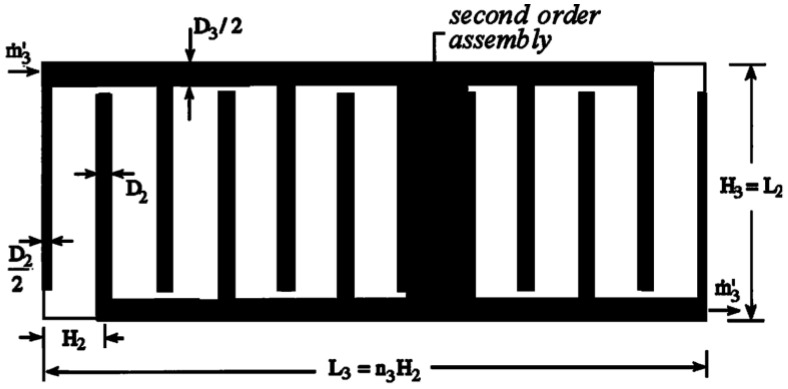
Third-order assembly with constant channels [74].

**Figure 8:**
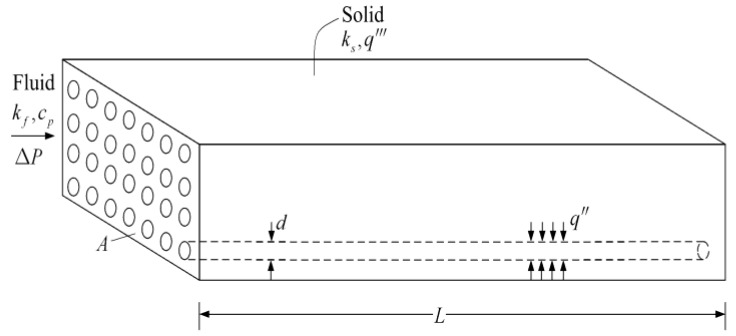
Model of heat-generating volume [75].

**Figure 9:**
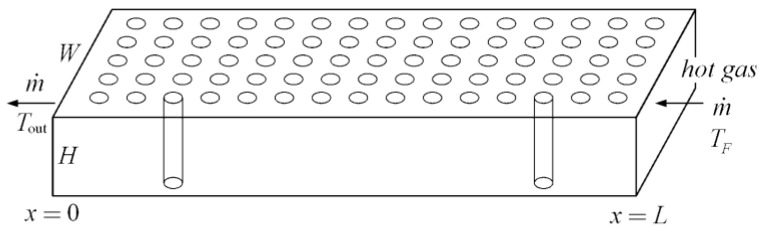
Model of a steam generator [76].

Constant cross-sectional channels in a rectangular heat generating body are shown in Figure 7 [74] where $D_{2}$, $D_{3}$, $H_{2}$, $H_{3}$, $L_{2}$, and $L_{3}$ are the cooling channel widths, assembly widths, and assembly lengths of the second- and third-order assemblies, respectively, and $\dot{m}\prime_{3}$ is the mass flow rate. The heat generated in the body is cooled by the cooling channels and the volumes of the cooling channels and rectangular body are fixed. The minimizations of EDR and flow resistance were taken as the optimization objectives, respectively. Constructal optimization of the rectangular body was implemented and an optimal construct of the rectangular body was obtained. The obtained results were compared with those obtained based on MTD minimization. Similar to the work on the rectangular body, the cooling channel problem in a cuboid heat generating body (Figure 8) [75] was investigated. In Figure 8, $k_{s}$, $k_{f}$, $c_{p}$, q‴, ΔP, A, d, and L are the thermal conductivities of the solid and fluid, specific heat at constant pressure, volumetric heat generation rate, fluid pressure drop, cross-sectional area, cooling channel diameter, and body length, respectively. Moreover, constructal optimization of the steam generator was also implemented based on EDR maximization (Figure 9) [76]. In Figure 9, $T_{F}$, $T_{out}$, $\dot{m}$, W, H, and L are the gas temperatures at the inlet and outlet, gas flow rate, width, height, and length of the steam generator, respectively.

**Figure 10:**
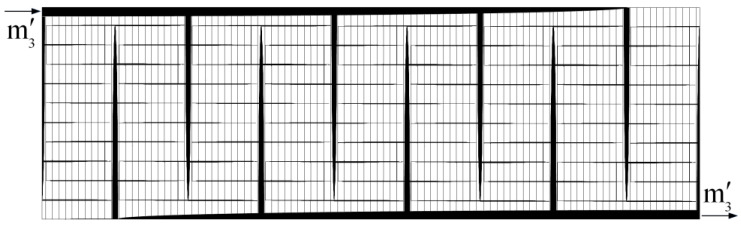
Third-order assembly with tapered channels [74].

**Figure 11:**
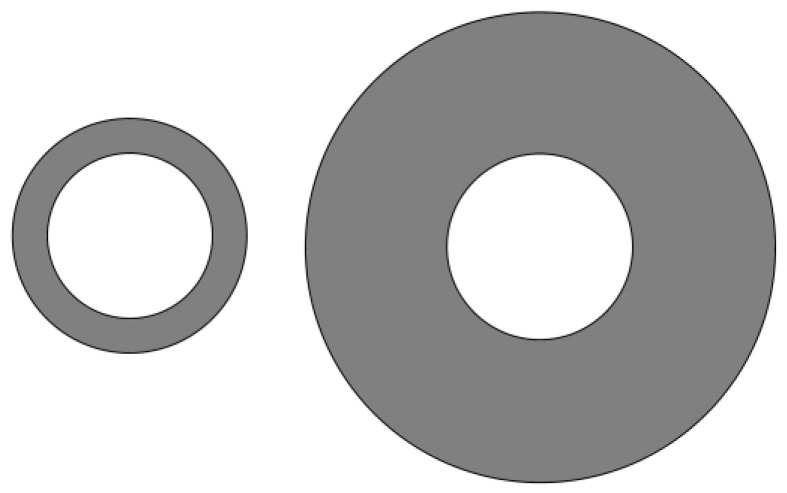
Optimal constructs based on the two optimization objectives [75].

**Figure 12:**
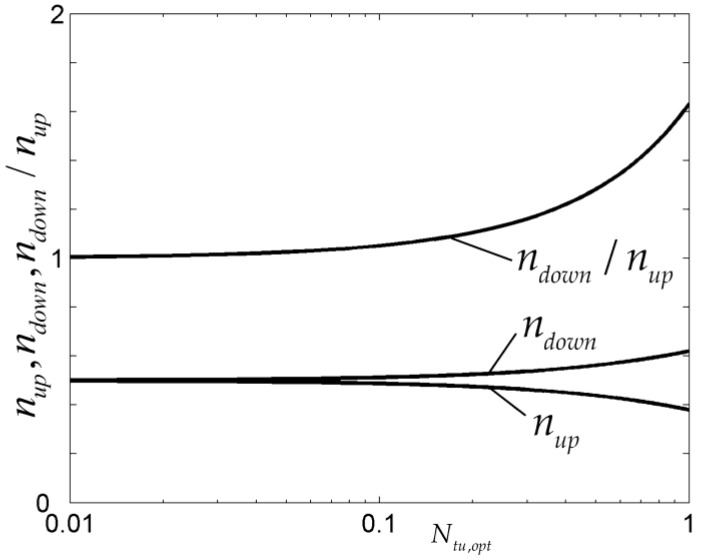
Number of riser and downcomer tubes [76].

The results showed that the optimal constructs of cooling channels in rectangular and cuboid bodies based on the minimizations of EDR and MTD, as well as those of a steam generator based on maximizations of EDR and HTR, are different. For the cooling channels in the rectangular body, the flow resistance is evidently reduced when EDR minimization of the rectangular element is conducted, and it can be further reduced by adopting variable cross-sectional channels (Figure 10). In Figure 10, $m\prime_{3}$ is the mass flow rate in the cooling channel of a third-order assembly. For the cooling channels in the cuboid, an optimal volume fraction of cooling channels exists when EDR minimization is conducted but it does not exist in the MTD minimization. Compared with the optimal results obtained by MTD minimization (Figure 11), the ETR obtained by EDR minimization is reduced by 23.12% and the global HTP of heat generation body is improved. For the steam generator, the optimal number of downcomer tubes is larger than that of riser tubes (Figure 12) and they locate at the same order of magnitude. In Figure 12, $n_{up}$, $n_{down}$, and $N_{tu,opt}$ are the numbers of riser and downcomer tubes and the optimal number of heat transfer units, respectively. The average HTR obtained by EDR maximization is increased by 58.7% compared with that obtained by HTR maximization, which illustrates an evident improvement of the global HTP.

### 2.3. Porous Medium Mass Transfer Problems

The mass transfer problem has many similarities with the heat conduction problem, and some scholars have conducted mass transfer constructal optimizations [77,78] by analogy with heat transfer constructal optimizations. Bejan and Errera [79] built a mass transfer model in a rectangular porous body and minimized its flow resistance by varying the internal and external shapes of the body. Bejan [80] and Errera and Bejan [81] further considered two- and three-dimensional mass transfer models and obtained more actual mass transfer performances compared with that derived in Reference [79]. Feng et al. [82] built a mass transfer model in a triangular porous body and found that the mass transfer performance was not always improved by increasing the complexity of the internal structure. Feng et al. [83] and Chen et al. [84] further built a cylindrical mass transfer model in a porous medium and obtained different optimal constructs of cylindrical models based on minimizations of maximum pressure drop (MPD) and mass EDR.

**Figure 13:**
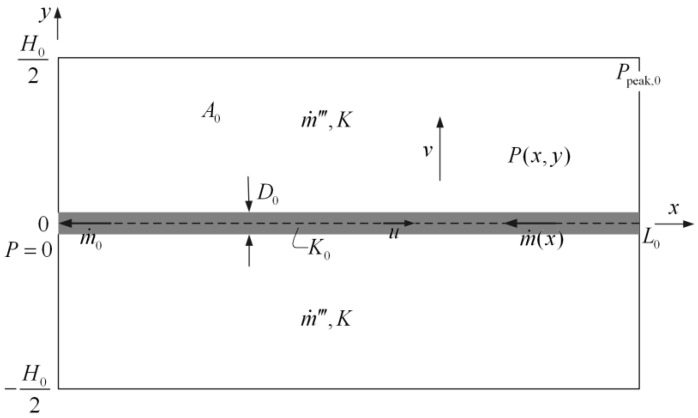
Mass transfer model in a rectangular element [86].

**Figure 14:**
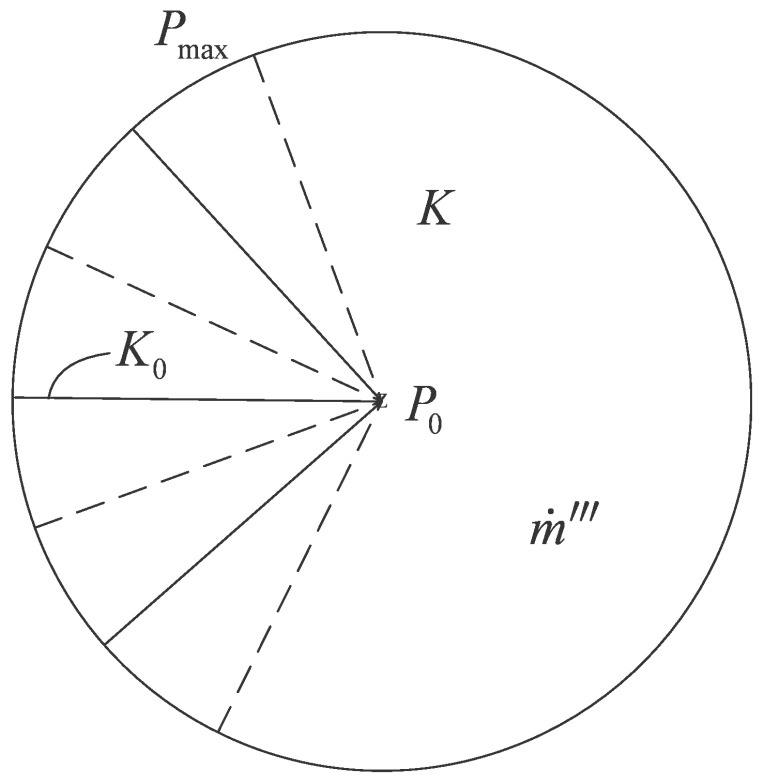
Mass transfer model in a radial-patterned disc [86].

A mass transfer model in a rectangular assembly is shown in Figure 13 [85,86] where P, $P_{\mathrm{peak},0}$, $\dot{m}\prime \prime \prime$, ${\dot{m}}_{0}$, K, $K_{0}$, u, v, $D_{0}$, $H_{0}$, $L_{0}$, and $A_{0}$ are the local pressure, maximum pressure, volumetric mass flow rate, total mass flow rate, permeabilities in the mass generation area and high permeability pathway (HPP), volume-averaged velocities in the *x* and *y* directions, HPP width, and width, length, and area of the rectangular element, respectively. The mass flow uniformly generates in the assembly and flows out from the left side of the HPP. The total areas of the assembly and HPP are fixed. The minimizations of MPD and mass EDR were taken as optimization objectives, respectively. Constructal optimizations of the assemblies with the premise of an optimized last-order construct and without this premise were implemented by varying their shapes. Comparisons of the constructal results with constant and tapered HPPs and the results based on the two optimization objectives were also conducted. Similar to the work of the rectangular assembly, the “disc-to-point” mass transfer problem with HPP (Figure 14) [86] was also investigated based on the constructal theory. In Figure 14, $\dot{m}\prime \prime \prime$, $P_{0}$, $P_{\max}$, K, and $K_{0}$ are the volumetric mass flow rate, outlet pressure, maximum pressure, and the permeabilities in the mass generation area and HPP, respectively.

**Figure 15:**
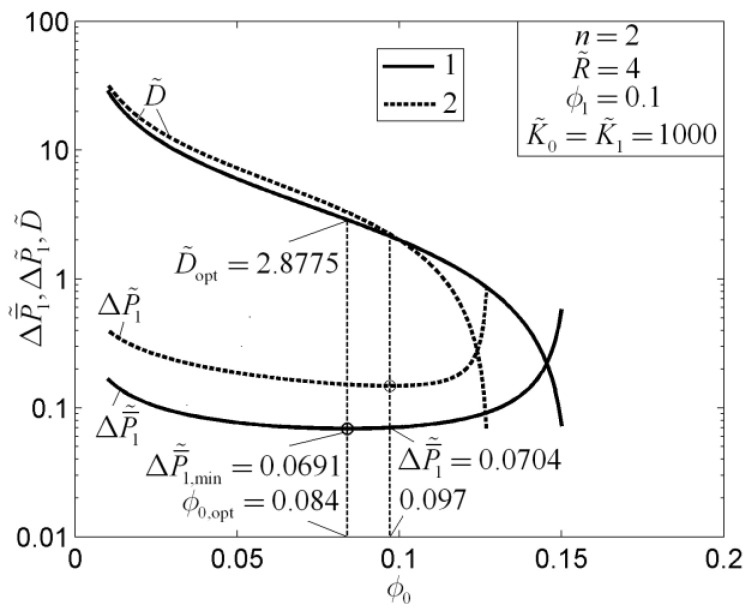
Optimal results with different optimization objectives [86].

**Figure 16:**
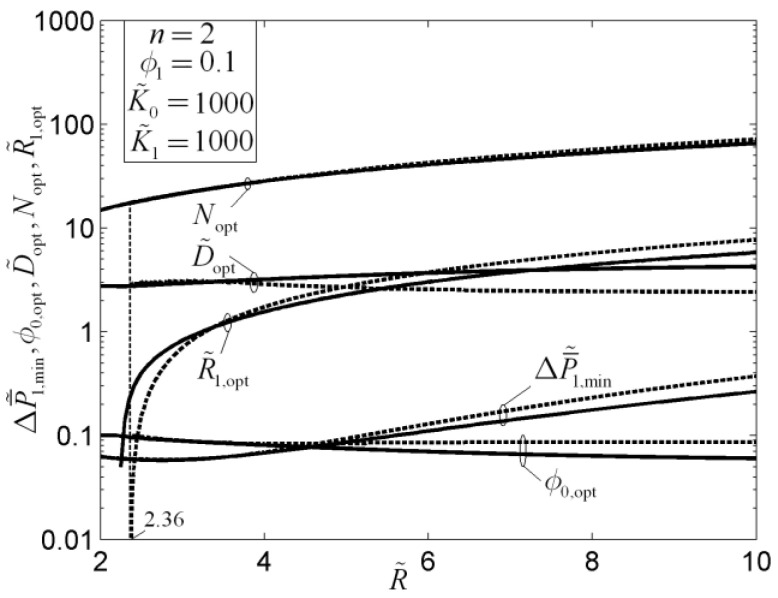
Effect of a dimensionless radiuson the optimal construct [86].

The results showed that the optimal constructs of a rectangular mass transfer assembly with tapered HPP obtained by the minimizations of MPD and mass EDR are different. Compared with the optimal results obtained by MPD minimization, the shape of the element obtained by mass EDR minimization is slender, the element number in the first-order assembly is larger, and the average pressure drop (APD) is lower. Compared with the rectangular mass transfer assembly with constant HPP, the MPDs of the element and first-order assembly with tapered HPPs are decreased by 6% and 11%, respectively, and the mass transfer performances (MTPs) of the assemblies are improved. When the premise of an optimized last-order construct is adopted, the optimal constructs of disc-shaped mass transfer assembly obtained based on the minimizations of MPD and mass EDR are different (Figure 15). In Figure 15, n, $\tilde{R}$, $\phi_{0}$, $\phi_{1}$, ${\tilde{K}}_{0}$, ${\tilde{K}}_{1}$, $\tilde{D}$, $\Delta {\tilde{P}}_{1}$, and $\Delta {\tilde{\bar{P}}}_{1}$ are the branch number, dimensionless radius of a first-order disc, elemental and first-order volume fractions of HPPs, permeability ratios of HPPs, width ratio of elemental and first-order HPPs, dimensionless maximum and average pressure drops of a first-order disc, respectively. The critical radius of the latter construct is smaller than that of the former. The latter construct reduces the APD of the disc-shaped assembly evidently, which reflects the essential requirement of MTP optimization. Moreover, the APD can be further decreased by releasing the premise of an optimized last-order construct (Figure 16). In Figure 16, $\phi_{0,opt}$, $N_{opt}$, ${\tilde{D}}_{opt}$, ${\tilde{R}}_{1,opt}$, and $\Delta {\tilde{\bar{P}}}_{1,\min}$ are the optimal elemental volume fraction of HPP, number of elemental sectors, width ratio of elemental and first-order HPPs, dimensionless radius of the central disc, and minimum dimensionless average pressure drop of a first-order disc, respectively.

## 3. Conclusions

Heat and mass transfer enhancements exist widely in engineering problems and the constructal theory has become a useful tool to solve these problems. The optimizations based on maximum potential difference minimization can only reflect the local transfer performances of transfer bodies. To improve the global transfer performances of transfer bodies, a series of constructal optimizations were conducted by combing the entransy theory with the constructal theory. This paper reviews constructal optimizations, performed at the Naval University of Engineering, for heat and mass transfers based on EDEP. Optimal constructs of the heat conduction and finned cooling bodies, cooling channel bodies, steam generators, and porous medium mass transfer bodies were obtained by optimizing their external and internal structures. Comparisons of the optimal results obtained by different optimization objectives and assembly methods were conducted. The results show the following:

(1) For the heat conduction and finned cooling problems, the MHTTD of the tapered assembly is not always decreased when the internal complexity of the assembly increases, but there exists an optimal order of the assembly that leads to the minimum MHTTD. The critical radii of the disc-shaped assembly based on the minimizations of EDR and MTD, which determine whether the radial-patterned disc or tree-shaped disc is adopted, are different. The HCP of the disc-shaped assembly can be further improved by releasing the premise of an optimized last-order construct. The global HTPs of Y-shaped and tree-shaped fins are better than that of a T-shaped fin. The global HTP of a tree-shaped fin is not always better when the internal structure becomes more complex.
(2) For the cooling channel and steam generator problems, the optimal construct of cooling channels in rectangular and cuboid bodies based on the minimizations of EDR and MTD, as well as those of a steam generator based on the maximizations of EDR and HTR, are different. The flow resistance of the cooling channels in the rectangular body is evidently reduced when EDR minimization of the rectangular element is conducted, and it can be further reduced by adopting variable cross-sectional channels. There exists an optimal volume fraction of cooling channels in the cuboid when EDR minimization is conducted, but it does not exist in the MTD minimization. Compared with the optimal results obtained by MTD minimization, the ETR of the cuboid body obtained by EDR minimization is reduced by 23.12% and its global HTP is improved. The average HTR of a steam generator obtained by EDR maximization is increased by 58.7% compared with that by HTR maximization, which illustrates an evident improvement of the global HTP.
(3) For the porous medium mass transfer problems, the optimal constructs of rectangular mass transfer assembly with tapered HPP obtained by the minimizations of MPD and mass EDR are different. Compared with the rectangular mass transfer assembly with constant HPP, the MPDs of the element and first-order assembly with tapered HPPs are decreased by 6% and 11%, respectively, and the MTPs of the assemblies are improved. When the premise of an optimized last-order construct is adopted, the optimal constructs of a disc-shaped mass transfer assembly obtained based on the minimizations of MPD and mass EDR are different. The latter construct reduces the APD of the disc-shaped assembly evidently, which reflects the essential requirement of MTP optimization. Moreover, the APD can be further decreased by releasing the premise of an optimized last-order construct.

The constructal optimizations for heat and mass transfers based on EDEP make the global HTPs and MTPs of the transfer systems effectively improve. These optimizations show the merit of the entransy theory and illustrate the significance of entransy optimization. The corresponding results reviewed in this paper can provide new guidelines for the designs of heat and mass transfer systems.
